# Sarcopenia and Systemic Inflammation Response Index Predict Response to Systemic Therapy for Hepatocellular Carcinoma and Are Associated With Immune Cells

**DOI:** 10.3389/fonc.2022.854096

**Published:** 2022-04-08

**Authors:** Man Zhao, Xiaoling Duan, Xin Han, Jinfeng Wang, Guangjie Han, Lili Mi, Jianfei Shi, Ning Li, Xiaolei Yin, Jiaojiao Hou, Fei Yin

**Affiliations:** Department of Gastroenterology, The Fourth Hospital of Hebei Medical University, Shijiazhuang, China

**Keywords:** hepatocellular carcinoma, sarcopenia, psoas muscle index, systemic inflammatory response index, immune cell

## Abstract

**Background:**

Systemic therapies, including immune checkpoint inhibitors (ICIs) and tyrosine kinase inhibitors (TKIs), have challenged the use of conventional therapies for hepatocellular carcinoma (HCC). It is crucial to determine which patients could benefit most from combination therapy. This study aims to examine the associations of sarcopenia and systemic inflammation response index (SIRI) with the treatment responses and efficacies in patients with HCC treated with ICIs and tyrosine kinase inhibitors TKIs, as well as investigate the correlation between sarcopenia and inflammatory or immune states.

**Methods:**

We reviewed 160 patients with HCC treated with TKIs and ICIs. The patients’ psoas muscle size was measured on axial computed tomography scans and normalized for the patients’ height squared. This value was referred to as the psoas muscle index (PMI). Sarcopenia was determined from PMI and their relationships with patients’ clinicopathological characteristics, inflammation indexes, peripheral blood T-cell subsets and survival were evaluated.

**Results:**

Sarcopenia and systemic inflammation response index (SIRI) were independent predictors for overall survival and progression-free survival. Patients with high PMI and low SIRI demonstrated significantly better median overall survival and progression-free survival (36.0 months and 9.6 months, respectively) than those with either low PMI or high SIRI (20.8 months and 6.0 months, respectively) and those with both high SIRI and low PMI (18.6 months and 3.0 months, respectively). Portal vein tumor thrombus (P=0.003), eastern cooperative oncology group performance status score of 1 (P=0.048), high alkaline phosphatase (P=0.037), high neutrophil-to-lymphocyte ratio (NLR) (P=0.012), low lymphocyte-to-monocyte ratio (LMR) (P=0.031), high platelet-to-lymphocyte ratio (PLR) (P=0.022) and high SIRI (P=0.012) were closely associated with an increased incidence of sarcopenia. PMI was negatively correlated with SIRI (r = -0.175, P=0.003), NLR (r = -0.169, P=0.036), and PLR (r = -0.328, P=0.000) and was significantly positively correlated with LMR (r = 0.232, P=0.004). The CD3+ and CD4+ T-cell counts of the high PMI group were significantly higher than those of the low PMI group.

**Conclusion:**

Sarcopenia and high SIRI were associated with reduced survival in patients with HCC treated with ICIs and TKIs. Sarcopenia could affect inflammatory states and the immune microenvironment.

## Introduction

Hepatocellular carcinoma (HCC) is one of the leading causes of cancer-related death; however, a limited number of systemic treatment options for advanced HCC exist. Currently, systemic therapies, including immune checkpoint inhibitors (ICIs) and tyrosine kinase inhibitors (TKIs), have challenged the use of conventional therapies for HCC. The field has witnessed substantial progress in the development of systemic therapies in the past 5 years, with studies reporting a marked increase in overall survival and in the quality of life of patients (1). For example, the natural history of advanced-stage HCC cases involves a median survival of 8 months and the approved combination of atezolizumab (anti-PDL1 antibody) and bevacizumab (anti-VEGF antibody) has more than doubled this life expectancy and improved the patient-reported outcomes (2). Although their clinical benefit is apparent, the use of ICIs and TKIs is limited owing to the associated cost. It is crucial to explore effective biomarkers for identifying patients who may benefit from combination therapy.

Sarcopenia is a progressive and generalized skeletal muscle disease characterized by accelerated loss of muscle mass and function (3). The disease has been associated with higher mortality among patients with cancer (4, 5). Sarcopenia has a negative effect on the body composition and can damage the body’s immune system. For patients receiving targeted therapy and immunotherapy, the immune state of the body determines treatment response and efficacy, so sarcopenia is being recognized as increasingly important for predicting tumor prognosis and therapeutic response.

Immunity and inflammation are basic features of the tumor microenvironment. A host’ s inflammatory and immune response to a tumor leads to the up- or downregulation of tumor proliferation and metastasis (6). There is increasing evidence that inflammation indexes can be employed to predict the prognosis of patients with cancer. The systemic inflammation response index (SIRI), neutrophil-to-lymphocyte ratio (NLR), platelet-to-lymphocyte ratio (PLR) and lymphocyte-to-monocyte ratio (LMR) are widely studied markers that have been proven effective in predicting patient survival in various kinds of cancer (7–10). Peripheral blood T-cell subsets are effective in reflecting the systemic immune status (11, 12). For example, CD8+ T-cells are essential immunological determinants for HBV-related HCC prognosis (13). Systematic analysis of the relationship between sarcopenia and the inflammatory indexes or immune cells would add greatly to our understanding of their role in tumor progression. Thus, the aim of this study was to investigate the role of sarcopenia as a predictor and the relationship between sarcopenia and systemic inflammation and immune status in patients with HCC.

## Patients and Methods

### Patients and Treatments

We retrospectively enrolled HCC patients who received TKIs and ICIs from January 2018 to December 2020 at the Fourth Hospital of Hebei Medical University.

The inclusion criteria were as follows: 1) age ≥18 years; 2) histologically confirmed HCC or a clinical diagnosis based on dynamic imaging and an underlying chronic liver disease; 3) patients who were not eligible for radical treatments, such as surgery and ablation; 4) patients who had not previously taken any systemic treatment for HCC; 5) TKI in combination with ICI as first-line treatment; 6) undergoing at least one cycle of systematic treatment; 7) patients with an eastern cooperative oncology group performance status (ECOG PS) of 0-1; 8) stage B or C categorization based on the Barcelona Clinic Liver Cancer (BCLC) staging system; 9) Child-Pugh A or B; 10) patients with at least one measurable target lesion; 11) patients with available cross-sectional abdominal images with computed tomographic (CT) scans within 1 months before systematic treatment. The exclusion criteria were as follows: 1) patients with combined immune and endocrine system diseases; 2) patients with cooccurrence of other lymphatic system disorders or malignant hematologic diseases, renal and/or hepatic failure, or systematic inflammatory diseases; 3) patients with a history of malignant tumors in other organs and liver metastasis; 4) patients with concurrent hepatitis A, hepatitis E, or human immunodeficiency virus infection.

Treatment options included lenvatinib combined with pembrolizumab/nivolumab/sintilimab/camrelizumab and sorafenib combined with sintilimab/camrelizumab. The dosing of the drugs is as follows: 1) lenvatinib 12 mg (if bodyweight ≥60 kg) or 8 mg (if bodyweight <60 kg) orally once daily; 2) sorafenib starting at 200 mg orally twice daily, with subsequent dose increase to 400 mg twice daily if it is well-tolerated); 3) pembrolizumab 200 mg intravenously every 3 weeks; 4) nivolumab 240 mg intravenously every 2 weeks; 5) camrelizumab 200 mg (for bodyweight ≥50 kg) or 3 mg/kg (for bodyweight <50 kg) intravenously every 2 weeks; and 5) sintilimab 200 mg intravenously every 3 weeks.

### Data Collection

Clinical information was retrieved from electronic medical records. Baseline patient characteristics, including demographics, etiology, presence of cirrhosis, Eastern Cooperative Oncology Group-performance status (ECOG-PS), Child–Pugh Class score, tumor markers, routine blood test results, liver function parameters, peripheral blood T-cell subsets, imaging examination and treatment history were examined.

### Systemic Inflammatory Index

Routine blood results were collected within 1 week before treatment, and the SIRI, NLR, PLR and LMR were calculated. The calculations were as follows: SIRI = neutrophil count × monocyte/lymphocyte count; NLR = neutrophil count/lymphocyte count; PLR = platelet count/lymphocyte count; and LMR = lymphocyte count/monocyte count.

### Assessment of Sarcopenia and PMI

**Figure 1:**
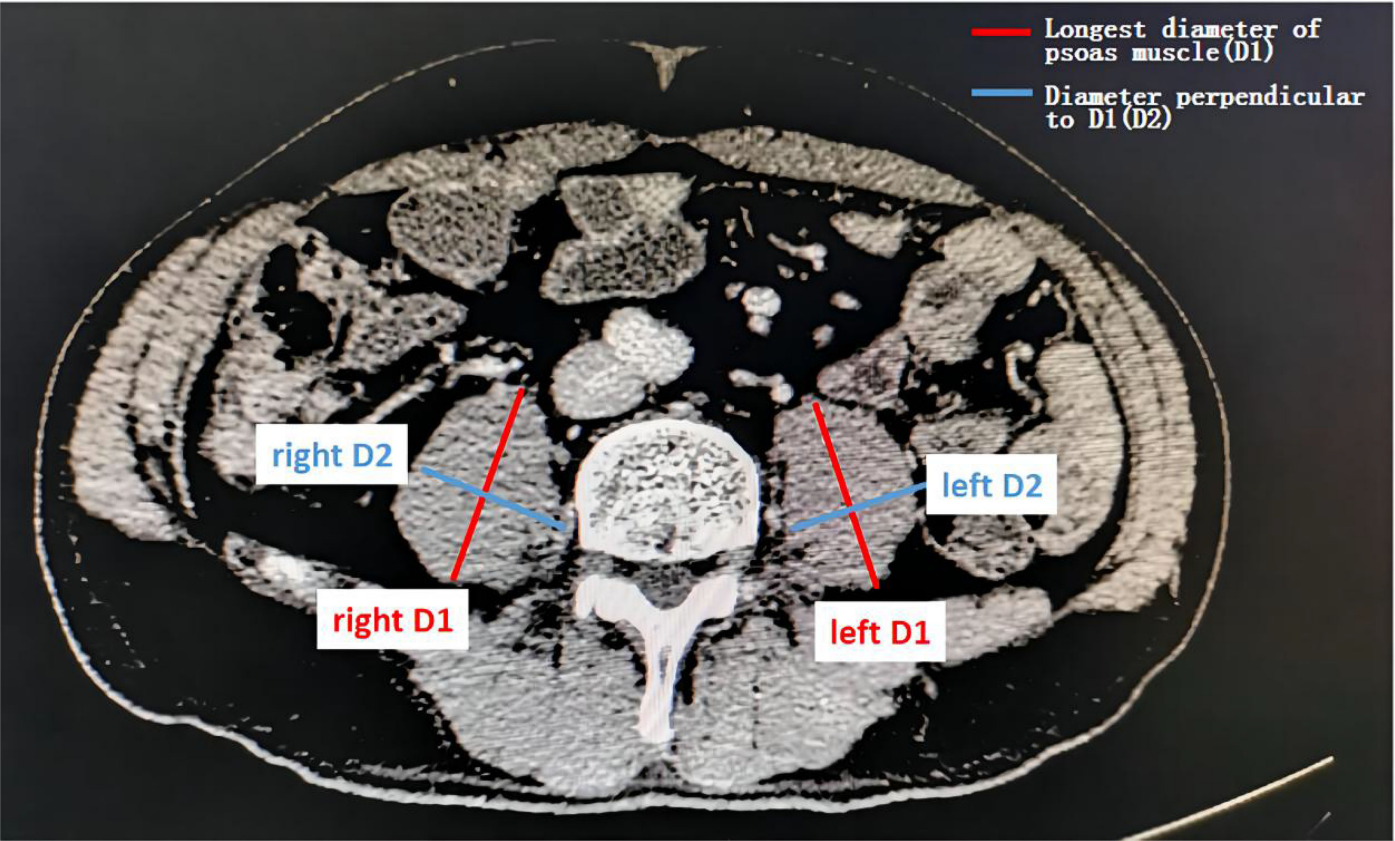
Assessment of the psoas muscle index (PMI). Sarcopenia was assessed by measuring the longest diameter (D1) and the perpendicular diameter (D2) of the right (ri) and left (le) psoas muscle on an axial computed tomography (CT) scan in the same plane and normalizing it for the patients’ height squared. This value is referred to as the psoas muscle index (PMI).

Sarcopenia was assessed by measuring the longest diameter (D1) and the perpendicular diameter (D2) of the right (ri) and left (le) psoas muscle on an axial CT scan. All diameters were measured in the same CT plane, which was usually between lumbar vertebral body (LVB) 3 and LVB 4 (14). An example image of the psoas muscle measurement is displayed in **Figure 1**. Psoas muscle index (PMI) was calculated as follows:


$$\frac{PMI=\left[mm/m^{2}\right]=\left(riD1\left[mm\right]+riD2\left[mm\right]+leD1\left[mm\right]+leD2\left[mm\right]\right)/4}{Patient'sheight{\left[m\right]}^{2}}$$

PMI is an effective proxy for sarcopenia. The CT images were provided by trained radiologists.

### Follow-Up

All patients were assessed every 1-2 months with radiologic and laboratory evaluations. The last follow-up date was November 2021. OS was defined as the interval between the first day of therapy and the date of death or the date of the last follow-up. PFS was defined as the interval between the first day of therapy and the date of disease progression or the date of death. Radiological responses were defined using the response evaluation criteria in solid tumors v1.1. Disease control rate and overall response rate were determined by the best radiologic response after CKI and ICI treatment: disease control rate included complete response, partial response, and stable disease; overall response rate included complete and partial responses, respectively.

### Statistical Analysis

Survival analysis was performed using the Kaplan−Meier method. The differences between the survival curves were compared by the log rank test. Multivariate Cox hazard regression analysis was performed on the factors that were shown to be significant on univariate analysis. The best cutoff values were determined by receiver operating characteristic (ROC) curve analysis. Spearman correlation analysis was used to detect linear correlations. The significance level was set at 5%. All statistical data were generated using SPSS software 26.0.

## Results

### Baseline Characteristics of Patients

The current study included a total of 160 subjects. The median age of the patients was 58 years (range: 26−86 years); 129 (80.6%) patients were male; 143 (89.4%) patients had hepatitis B virus infection; 132 (82.5%) patients exceeded the up-to-seven criteria; and 45 (28.1%) patients had portal vein tumor thrombus (PVTT). Cirrhosis was present in 114 (71.3%) patients. There were 106 (67.9%) patients with barcelona clinic liver cancer (BCLC) stage C disease. The patients were in good physical condition; 99 (61.9%) patients had an ECOG-PS score of 0 **(** **Table 1** **)**.

**Table 1:** Baseline characteristics of patients stratified by sarcopenia.

Characteristics	Total(n=160)	Sarcopenia(n=120)	No sarcopenia(n=40)	P
Age				0.201
<58 years	82(51.3%)	58	24	
≥58 years	78(48.7%)	62	16	
Gender				0.083
Male	129(80.6%)	93	36	
Female	31(19.4%)	27	4	
Up-to-seven				0.118
>7	132(82.5%)	102	30	
≤ 7	37(17.5%)	17	10	
PVTT				**0.003**
Yes	45(28.1%)	41	4	
No	115(71.9%)	79	36	
ALP				**0.037**
>125U/L	58(36.3%)	49	9	
≤ 125U/L	102(63.7%)	71	31	
Liver cirrhosis				0.840
Yes	114(71.3%)	86	28	
No	46(28.7%)	34	12	
BCLC				0.177
B	54(32.1%)	37	17	
C	106(67.9%)	83	23	
ECOG-PS				**0.048**
0	99(61.9%)	69	30	
1	61(38.1%)	51	10	
AFP				0.360
>400ng/mL	74(46.3%)	58	16	
≤ 400ng/mL	86(53.7%)	62	24	
ALT				0.232
>50U/L	48(30.0%)	39	9	
≤ 50U/L	112(70.0%)	81	31	
Albumin				0.052
<35g/L	93(58.1%)	75	18	
≥ 35g/L	67(41.9%)	45	22	
NLR				**0.012**
≥ 3.25	95(59.4%)	78	17	
<3.25	65(40.6%)	42	23	
LMR				**0.031**
≥ 3.59	61(38.1%)	40	21	
<3.59	99(61.9%)	80	19	
PLR				**0.022**
≥ 145.25	79(49.4%)	53	26	
<145.25	81(50.6%)	67	14	
SIRI				**0.012**
≥ 1.64	67(41.9%)	57	10	
<1.64	93(58.1%)	63	30	

PVTT, portal vein tumor thrombus; ALP, alkaline phosphatase; BCLC, barcelona clinic liver cancer; ECOG-PS, eastern cooperative oncology group-performance status; AFP, alpha-fetoprotein; ALT, alanine transaminase; SIRI, systemic inflammation response index; NLR, neutrophil-to-lymphocyte ratio; PLR, platelet-to-lymphocyte ratio; LMR, lymphocyte-to-monocyte ratio.

Bold values means there is a statistically difference in the result.

### Optimal Cut - Off Analysis

**Figure 2:**
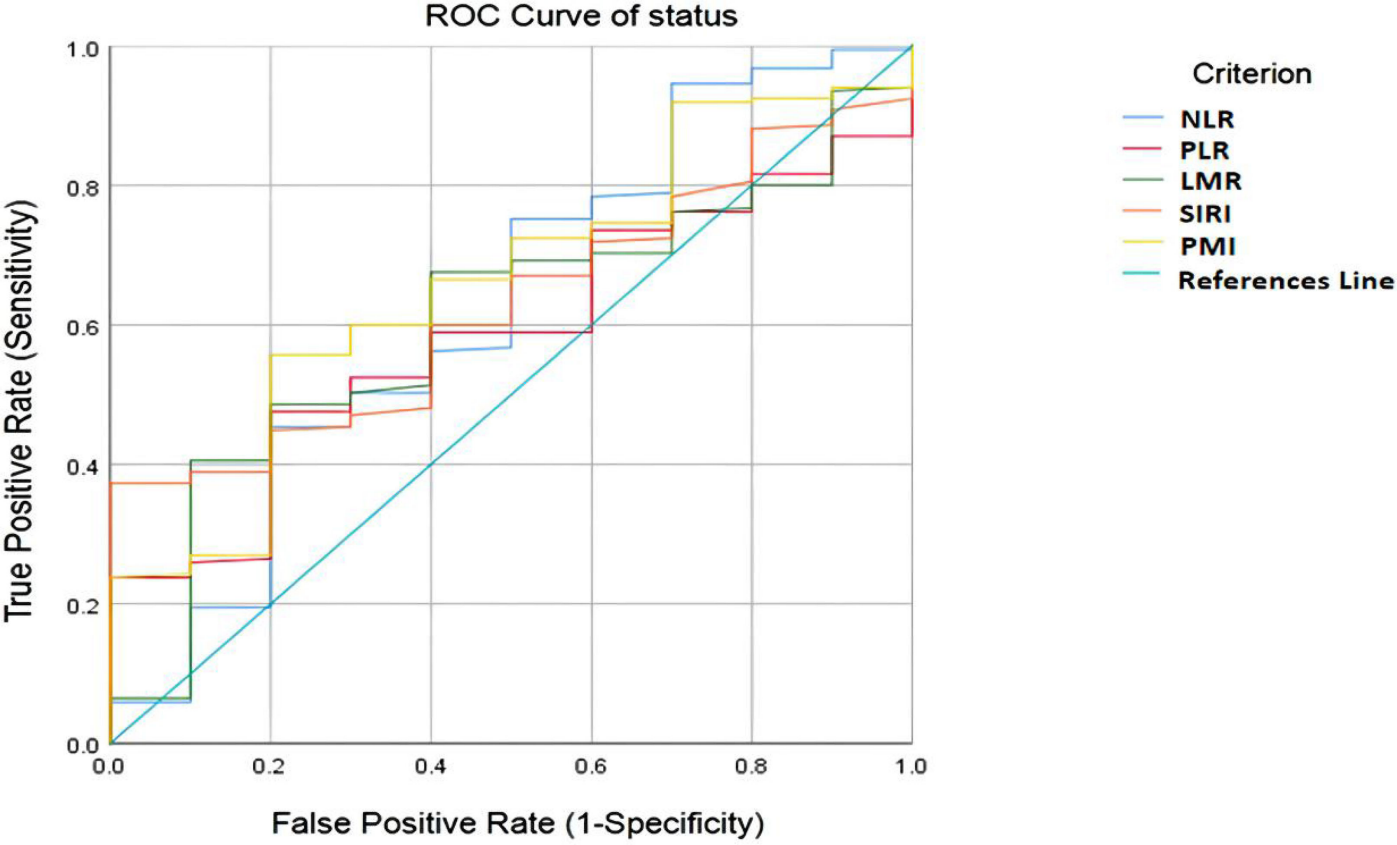
ROC curve analysis for optimal cut-off value of NLR, PLR, LMR, SIRI and PMI. ROC, receiver operating characteristic; NLR, neutrophil-to-lymphocyte ratio; PLR, platelet-to-lymphocyte ratio; LMR, lymphocyte-to-monocyte ratio; SIRI, systemic inflammation response index; PMI, psoas muscle index.

The optimal cutoff value for the patients was determined by ROC curve analysis as follows: NLR=3.25, PLR = 145.25, LMR = 3.59, SIRI =1.64 and PMI=14.19. The area under the receiver operating characteristic curve values for NLR, PLR, LMR, SIRI and PMI in disease control prediction were 0.622, 0.586, 0.604, 0.627, and 0.659, respectively **(** **Figure 2** **)**.

### The Effect of SIRI and PMI on OS and PFS

**Figure 3:**
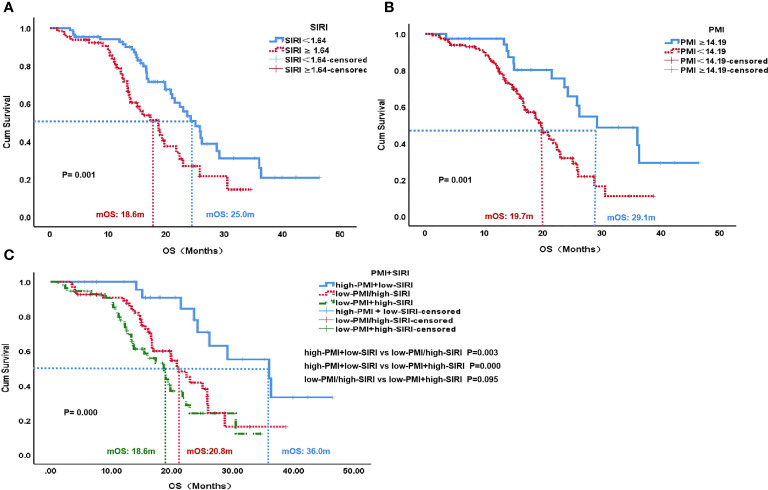
OS according to SIRI status **(A)**, PMI status **(B)** and PMI+SIRI status **(C)** in the patients with HCC. OS, overall survival; SIRI, systemic inflammation response index; PMI, psoas muscle index.

The median OS of the low SIRI group was 25.0 months, which was significantly higher than the 18.6 months of the high SIRI group (P=0.001). The median OS of the high PMI group was 29.1 months, which was higher than the 19.7 months of the low PMI group (P=0.001). Patients with high PMI and low SIRI demonstrated significantly better median OS (36.0 months) than those with either low PMI or high SIRI (20.8 months) and those with both high SIRI and low PMI (18.6 months)(P=0.000) **(** **Figure 3** **)**.

**Figure 4:**
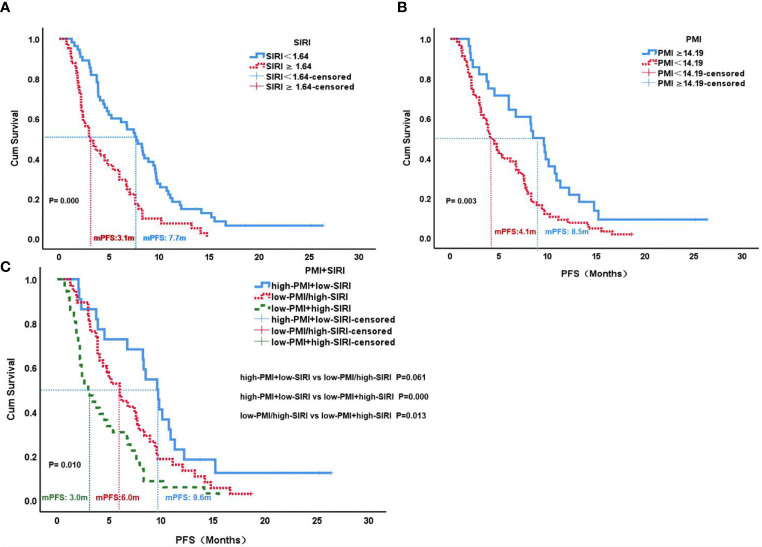
PFS according to SIRI status **(A)**, PMI status **(B)** and PMI+SIRI status **(C)** in the patients with HCC. PFS, progression-free survival; SIRI, systemic inflammation response index; PMI, psoas muscle index.

The median PFS of the low SIRI group was 7.7 months, which was significantly higher than the 3.1 months of the high SIRI group (P=0.000). The median PFS of the high PMI group was 8.5 months, which was significantly higher than the 4.1 months of the low PMI group (P=0.003). Patients with high PMI and low SIRI demonstrated better median PFS (9.6 months) than those with either low PMI or high SIRI (6.0 months) and those with both high SIRI and low PMI (3.0 months)(P=0.010) (**Figure 4**).

For the best response after treatment, the disease control rate and overall response rate for patients were 92.5% and 36.2%, respectively. However, the disease control rate (89.6% vs. 96.6%, P=0.816) and overall response rate (29.3% vs. 46.6%, P=0.272) of patients with low PMI and high SIRI were lower than those of patients with high PMI and low SIRI, but there was no statistical significance (**Table 2**).

**Table 2:** Tyrosine kinase inhibitors and immune checkpoint inhibitors efficacy results.

	N	DCR	P	ORR	P	CR	PR	SD	PD
		%		%		N (%)	N (%)	N (%)	N (%)
Overall	160	92.5		36.2		6(3.7)	52 (32.5)	90 (56.3)	12 (7.5)
PMI <14.19	120	91.7	0.892	33.4	0.373	2 (1.7)	38 (31.7)	70 (58.3)	10 (8.3)
PMI ≥14.19	40	95.0		45.0		4 (10.0)	14 (35.0)	20 (50.0)	2 (5.0)
SIRI ≥1.64	68	88.2	0.727	27.9	0.194	0 (0.0)	19 (27.9)	41 (60.3)	8 (11.8)
SIRI <1.64	92	95.7		42.4		6 (6.5)	33 (35.9)	49 (53.3)	4(4.3)
PMI+SIRI			0.122		0.540				
High PMI(≥14.19)+ Low SIRI(<1.64)	30	96.6		46.6		4 (13.3)	10 (33.3)	15 (50.0)	1 (3.4)
Low PMI(<14.19)/ High SIRI(≥1.64)	72	93.0		37.5		2 (2.8)	25(34.7)	40 (55.5)	5 (7.0)
Low PMI(<14.19)+ High SIRI(≥1.64)	58	89.6		29.3		0 (0.0)	17(29.3)	35 (60.3)	6 (10.4)

SIRI, systemic inflammation response index; PMI, psoas muscle index; DCR, disease control rate; CR, complete response; PR, partial response; SD, stable disease; ORR, overall response rate; PD, progressive disease.

### Multivariate Cox Regression Analyses for PFS and OS

In the multivariate Cox regression analysis, BCLC (HR, 1.576; 95% CI, 1.010−2.458; P=0.045), SIRI (HR, 1.817; 95% CI, 1.165−2.835; P=0.008) and PMI (HR, 1.757; 95% CI, 1.090−2.831; P=0.021) were independent predictors for PFS **(** **Table 3**). AFP (HR, 2.005; 95% CI, 1.251−3.213; P=0.004), SIRI(HR, 1.800; 95% CI, 1.117−2.901; P=0.016) and PMI (HR, 2.464; 95% CI, 1.308−4.642; P=0.005) were independent predictors for OS (**Table 4**). PMI and SIRI were independent risk factors for PFS and OS in patients with HCC.

**Table 3:** Prognostic factors for progression-free survival.

	PFS			
	Univariate		Multivariate	
	HR(95%CI)	P	HR(95%CI)	P
Age ≥58years vs 58< years	0.982(0.651—1.482)	0.932	—	—
Gender Male vs Female	1.073(0.639—1.801)	0.790	—	—
Uptoseven >7 VS ≤7	1.661(0.978—2.821)	0.061	—	—
PVTT yes vs no	1.250(0.735—2.124)	0.410	—	—
Extrahepatic metastasis yes vs no	1.431(0.927—2.208)	0.106	—	—
BCLC C vs B	1.870(1.217—2.873)	**0.004**	1.576(1.010—2.458)	**0.045**
ECOG-PS 1 vs 0	1.627(1.059—2.500)	**0.026**	—	—
AFP >400ng/mL vs ≤400ng/mL	1.458(0.965—2.202)	0.073	—	—
ALT >50U/L vs ≤50U/L	0.751(0.427—1.195)	0.227	—	—
ALP >125U/L vs ≤125U/L	1.242(0.791—1.950)	0.346	—	—
NLR ≥3.25 vs <3.25	1.668(1.099—2.531)	**0.016**	—	—
PLR ≥145.25 vs <145.25	1.500(0.982—2.291)	0.061	—	—
LMR <3.59 vs ≥3.59	0.667(0.436—1.022)	0.063	—	—
SIRI ≥1.64 vs <1.64	2.212(1.446—3.383)	**0.000**	1.817(1.165—2.835)	**0.008**
PMI <14.19 vs ≥14.19	1.988(1.246—3.171)	**0.004**	1.757(1.090—2.831)	**0.021**

PVTT, portal vein tumor thrombus; ALP, alkaline phosphatase; BCLC, barcelona clinic liver cancer; ECOG-PS, eastern cooperative oncology group-performance status; AFP, alpha-fetoprotein; ALT, alanine transaminase; SIRI, systemic inflammation response index; NLR, neutrophil-to-lymphocyte ratio; PLR, platelet-to-lymphocyte ratio; LMR, lymphocyte-to-monocyte ratio; PMI, psoas muscle index; PFS, progression-free survival. Bold values means there is a statistically difference in the result.

**Table 4:** Prognostic factors for overall survival.

	OS			
	Univariate		Multivariate	
	HR(95%CI)	P	HR(95%CI)	P
Age ≥58years vs 58< years	1.097(0.694—1.732)	0.693	—	—
Gender Male vs Female	1.071(0.596—1.924)	0.820	—	—
Uptoseven >7 VS ≤7	1.654(0.911—3.001)	0.098	—	—
PVTT yes vs no	1.725(1.059—2.808)	**0.028**	—	—
Extrahepatic metastasis yes vs no	1.026(0.639—1.647)	0.916	—	—
BCLC C vs B	1.733(1.060—2.832)	**0.028**	—	—
ECOG-PS 0 vs 1	1.205(0.750—1.936)	0.441		
AFP >400ng/mL vs ≤400ng/mL	1.952(1.228—3.102)	**0.005**	2.005(1.251—3.213)	**0.004**
ALT >50U/L vs ≤50U/L	1.147(0.684—1.921)	0.603	—	—
ALP >125U/L vs ≤125U/L	1.822(1.118—2.969)	**0.016**	—	—
NLR ≥3.25 vs <3.25	1.737(1.074—2.807)	**0.024**	—	—
PLR ≥145.25 vs <145.25	1.455(0.918—2.305)	0.110	—	—
LMR <3.59 vs ≥3.59	1.107(0.682—1.798)	0.681	—	—
SIRI ≥1.64 vs <1.64	2.034(1.274—3.248)	**0.003**	1.800(1.117—2.901)	**0.016**
PMI <14.19 vs ≥14.19	2.658(1.442—4.898)	**0.002**	2.464(1.308—4.642)	**0.005**

PVTT, portal vein tumor thrombus; ALP, alkaline phosphatase; BCLC, barcelona clinic liver cancer; ECOG-PS, eastern cooperative oncology group-performance status; AFP, alpha-fetoprotein; ALT, alanine transaminase; SIRI, systemic inflammation response index; NLR, neutrophil-to-lymphocyte ratio; PLR, platelet-to-lymphocyte ratio; LMR, lymphocyte-to-monocyte ratio; PMI, psoas muscle index; OS, overall survival. Bold values means there is a statistically difference in the result.

### The Relationship Between PMI, SIRI and Peripheral Blood T-Cell Subsets

**Figure 5:**
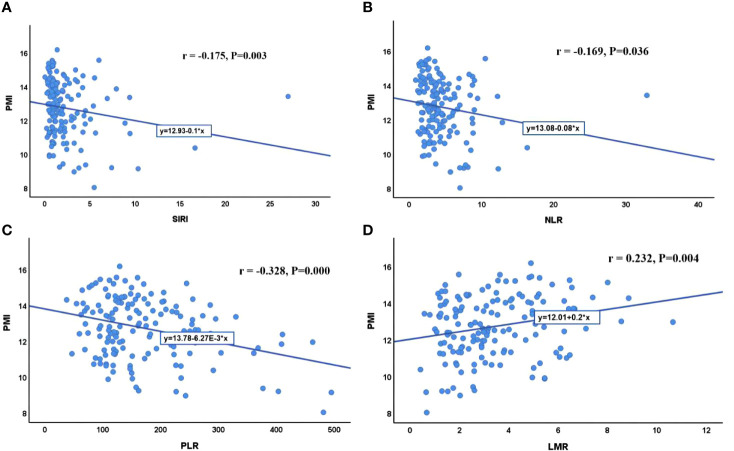
Correlation between PMI and inflammatory indicators. **(A)** Correlation between PMI and SIRI; **(B)** Correlation between PMI and NLR; **(C)** Correlation between PMI and PLR; **(D)** Correlation between PMI and LMR. SIRI, systemic inflammatory response index; NLR, neutrophil-to-lymphocyte ratio; PLR, platelet-to-lymphocyte ratio; LMR, lymphocyte-to-monocyte ratio; PMI, psoas muscle index.

With PVTT (P=0.003), ECOG-PS score of 1 (P=0.048), high ALP (P=0.037), high NLR (P=0.012), low LMR (P=0.031), high PLR (P=0.022) and high SIRI (P=0.012) were closely associated with an increased incidence of sarcopenia (**Table 1**). We further analyzed the correlation between PMI and inflammatory indicators. Our results showed that PMI was negatively correlated with SIRI (r = -0.175, P=0.003), NLR (r = -0.169, P=0.036) and PLR (r = -0.328, P=0.000) and was significantly positively correlated with LMR (r = 0.232, P=0.004) (**Figure 5**). Moreover, it had been recently discovered that PMI was closely related to peripheral blood T-cell counts. The CD3+ and CD4+ T-cell counts of the high PMI group were significantly higher than those of the low PMI group. The CD8+ T-cell counts and CD4+/CD8+ ratios of the high PMI group were also higher, but without any significant difference (**Table 5**).

**Table 5:** Peripheral blood T-cell subsets according to PMI.

Characteristics	CD3+T-cellcounts (mean)	P	CD4+T-cell counts(mean)	P	CD8+T-cell counts(mean)	P	CD4+/CD8+ ratio(mean)	P
PMI<14.19	0.449 × 10^9^	**0.023**	0.242 × 10^9^	**0.007**	0.183 × 10^9^	0.205	1.70	0.627
PMI ≥14.19	0.857 × 10^9^		0.443 × 10^9^		0.376 × 10^9^		1.91	

PMI, psoas muscle. Bold values means there is a statistically difference in the result.

## Discussion

When considering the limited response rate and utilization rate of TKIs and ICIs in patients with HCC, potential biomarkers for predicting treatment outcomes have attracted the attention of several physicians. This study comprehensively analyzed the predictive power of sarcopenia and inflammatory immune indicators for the treatment outcomes of patients with HCC treated with TKIs and ICIs.

Sarcopenia has been defined as the “progressive loss of muscle mass and strength with a risk of adverse outcomes such as disability, poor quality of life and death” (15). Recent studies have shown that muscle loss is associated with an impaired prognosis in patients with different solid tumors (16). In patients with HCC, sarcopenia has been associated with impaired OS and disease-free survival after surgical resection or radiofrequency ablation (17, 18). Most European and American studies have found that sarcopenia is an independent risk factor for the prognosis of HCC in patients undergoing surgical resection (19). Aliya et al. found that sarcopenia was associated with shorter survival (< 1 year) and less HCC necrosis (<50% necrosis or >50% viable tumor) with targeted therapy and was negatively associated with the efficacy of targeted therapy (20). Kim et al. examined 102 patients with HCC treated with nivolumab and reported that patients with sarcopenia had a shorter OS than those without sarcopenia (21). A meta-analysis of 2501 patients with solid tumors reported a negative correlation between sarcopenia and ICI efficacy. Besides, the predictive power of sarcopenia was consistent across tumor types, including HCC (22). However, studies on sarcopenia and treatment response in the Chinese HCC population treated with TKIs and ICIs are relatively scarce. This study found that patients with sarcopenia before TKIs and ICIs treatment were more likely to have disease progression and had shorter survival. Sarcopenia was an independent risk factor for OS and PFS in patients with HCC.

Systemic inflammation is an important promoter of the proliferation, invasion, and metastasis of malignant cells (23–25). Many inflammatory markers, such as SIRI, LMR, NLR, and PLR, have been associated with poor prognosis for various cancers (7–10, 26). SIRI is a simple noninvasive prognostic marker based on the counts of peripheral neutrophils, monocytes and lymphocytes. High SIRI is associated with poor prognosis and has been confirmed in a variety of cancers. Survival analysis of patients with HCC treated with radiofrequency ablation revealed that OS and recurrence-free survival (RFS) were significantly higher in patients with a low SIRI than in those with a high SIRI. In a multivariate analysis, SIRI was an independent predictor of RFS (27). A study of 194 patients reported that pretreatment peripheral blood SIRI was an independent predictor of tumor response and clinical outcomes in patients with HCC undergoing transcatheter arterial chemoembolization. Indeed, patients with high SIRI might have a poor prognosis (28). Another study demonstrated a correlation between SIRI(P = 0.002) and early postoperative recurrence in patients with HCC (29). Our study evaluated the relationship between four inflammatory indicators and the clinical outcomes of HCC treated with ICIs and TKIs and proved that patients with high SIRI have a poor survival.

Among these inflammatory markers, SIRI was the best independent predictor of OS and PFS. Sarcopenia and SIRI could be potential biomarkers of response to TKIs and ICIs therapy. The current study also showed that risk groups based on sarcopenia and SIRI at baseline could successfully predict survival outcomes. Patients with high PMI and low SIRI had significantly better outcomes than those with either low PMI or high SIRI and those with both high SIRI and low PMI.

Sarcopenia and chronic inflammatory status play a role in TKIs and ICIs resistance. This study also found that PMI was negatively correlated with SIRI, NLR and PLR and was significantly positively correlated with LMR. Patients with sarcopenia had increased levels of inflammatory markers, which supports the fact that sarcopenia refects the increased metabolic activity leading to systemic inflammation and muscle depletion (30). A possible mechanism is as follows: cytokines such as tumor necrosis factor and interleukin 6 are produced by tumor cells or surrounding cells and promote protein degradation and decreased synthesis. Tumor necrosis factor inhibits skeletal myocyte differentiation, promotes muscle atrophy, and contributes to insulin resistance by impairing the insulin signaling pathway. Interleukin 6 can further reduce muscle protein synthesis (31). Increases in inflammatory cytokines can also lead to insulin resistance and muscle wasting by activating the ubiquitin−proteasome proteolytic pathway, while muscle loss itself further exacerbates insulin resistance. Low-grade systemic inflammation caused by the tumor (and possibly exacerbated by obesity or insulin resistance) could drive local inflammation in the muscle. This effect, in turn, further contributes to systemic inflammation and muscle degradation (32).

Immunity and inflammation are essential characteristics of the tumor microenvironment. Immune-related cells in the immune microenvironment have an important influence on the occurrence and development of tumor (33). In patients with surgically resected HCC, high levels of both CD3+ and CD8+ T-cells were significantly related to a low rate of recurrence(P = 0.007) and a prolonged RFS (P = 0.002) (27). Sarcopenia is also closely related to the immune microenvironment. Several reports of patients with malignant melanoma or advanced lung cancer had demonstrated that patients with sarcopenia frequently had poor survival outcomes after ICIs (34–36). Because skeletal muscle cells express major histocompatibility complexes which stimulate T cells, loss of skeletal muscle may disrupt the homeostatic balance. Moreover, the drop in myokines, especially IL-15, disturbs the tight balance of different T-cell subsets (36, 37). In this study, peripheral blood T lymphocytes, especially CD3+ and CD4+ T-cell counts, were significantly reduced in patients with sarcopenia. Thus, changes in the myokine levels as a result of sarcopenia may affect the efficacy of TKIs and ICIs treatment, indicating the predictive value of sarcopenia in this therapy.

Our approach has a few limitations. First, as the definition of sarcopenia, various cutoff values have existed in previous reports, and the authors of those reports decided the cutoff value by sex. However, we decided our cutoff values irrespective of sex because there were too few female patients in our study. Second, owing to the limited number of patients, the peripheral blood T lymphocyte subsets analysis had a limited statistical power. The statistical significance with limited number of patients need to be interpreted with caution because the observed effect may not result from true biological effect. Third, this study did not evaluate the effect of sarcopenia on drug-related adverse reactions and quality of life. We will further evaluate the impact of sarcopenia on patient safety, and finally draw more convincing conclusion.

## Conclusion

This study established that sarcopenia and SIRI can successfully predict the therapeutic responses of patients with HCC receiving ICIs and TKIs. Sarcopenia can objectively reflect the physical condition, nutritional status, and immune status of patients, while SIRI can reflect the inflammatory state of the body. The combination of sarcopenia and SIRI could be used to identify patients with poor treatment tolerance and high risk of tumor immune escape, as well as those who would benefit from combination therapy. In addition, sarcopenia could affect the inflammatory status and immune microenvironment, and the underlying molecular mechanisms warrant further investigation.

## Data Availability Statement

The raw data supporting the conclusions of this article will be made available by the authors, without undue reservation.

## Ethics Statement

The studies involving human participants were reviewed and approved by Ethics Committee of The Fourth Hospital of Hebei Medical University. The patients/participants provided their written informed consent to participate in this study.

## Author Contributions

Conception and design, FY and MZ. Manuscript writing, MZ. Collection and assembly of data, XD, XH, JW, GH, LM, JS, NL, XY, and JH. Data analysis and interpretation, FY and MZ. All authors contributed to the article and approved the submitted version.

## Conflict of Interest

The authors declare that the research was conducted in the absence of any commercial or financial relationships that could be construed as a potential conflict of interest.

## Publisher’s Note

All claims expressed in this article are solely those of the authors and do not necessarily represent those of their affiliated organizations, or those of the publisher, the editors and the reviewers. Any product that may be evaluated in this article, or claim that may be made by its manufacturer, is not guaranteed or endorsed by the publisher.
